# Population imaging discrepancies between a genetically-encoded calcium indicator (GECI) versus a genetically-encoded voltage indicator (GEVI)

**DOI:** 10.1038/s41598-021-84651-6

**Published:** 2021-03-05

**Authors:** Mei Hong Zhu, Jinyoung Jang, Milena M. Milosevic, Srdjan D. Antic

**Affiliations:** 1 Institute for Systems Genomics, Department of Neuroscience, UConn School of Medicine, Farmington, CT 06030 USA; 2 Center for Laser Microscopy, Faculty of Biology, University of Belgrade, 11000 Belgrade, Serbia

**Keywords:** Biological techniques, Biophysics, Neuroscience

## Abstract

Genetically-encoded calcium indicators (GECIs) are essential for studying brain function, while voltage indicators (GEVIs) are slowly permeating neuroscience. Fundamentally, GECI and GEVI measure different things, but both are advertised as reporters of “neuronal activity”. We quantified the similarities and differences between calcium and voltage imaging modalities, in the context of population activity (without single-cell resolution) in brain slices. GECI optical signals showed 8–20 times better SNR than GEVI signals, but GECI signals attenuated more with distance from the stimulation site. We show the exact temporal discrepancy between calcium and voltage imaging modalities, and discuss the misleading aspects of GECI imaging. For example, population voltage signals already repolarized to the baseline (~ disappeared), while the GECI signals were still near maximum. The region-to-region propagation latencies, easily captured by GEVI imaging, are blurred in GECI imaging. Temporal summation of GECI signals is highly exaggerated, causing uniform voltage events produced by neuronal populations to appear with highly variable amplitudes in GECI population traces. Relative signal amplitudes in GECI recordings are thus misleading. In simultaneous recordings from multiple sites, the compound EPSP signals in cortical neuropil (population signals) are less distorted by GEVIs than by GECIs.

## Introduction

Electrical signal (a membrane potential transient), is the primary substrate of rapid information processing in the brain^1^, where across the population of available neurons in a volume of tissue, only a small minority is electrically active during maintenance of any single stimulus in working memory^1–4^. Optical measurements of membrane potential changes in many individual neurons are technically challenging^5,6^, so researchers regularly use optical transients from GCaMP-expressing neurons and they report this as “*neuronal activity*”, meaning: “*neuronal electrical activity*”. The GCaMP-based indicators do not detect membrane potential changes; instead they detect intracellular concentration of a free calcium ion in the neuronal cytosol^7^. The idea of using calcium concentrations for studying electrical signaling is plagued by several problems. First, large temporal discrepancies between calcium and voltage optical waveforms were documented by experiments in which calcium and voltage are imaged in the same neuron^8–11^. Tremendous nonlinear amplitude discrepancies between calcium and voltage optical signals regularly occur. For example, activation of dendritic NMDA receptors mediates negligible voltage changes, but disproportionally massive calcium changes^8,12^. In experiments utilizing simultaneous voltage-calcium measurements from the same neuron, subthreshold depolarizations are obvious in the voltage records and completely lost in the concurrent calcium optical signals^9,13^. Finally, cellular processes unrelated to electrical activity, or not directly related to electrical activity, often produce noticeable changes in calcium ion concentration^14–16^.

Knowing all advantages and limitations of an experimental method is essential for a meaningful interpretation of experimental data^17–19^, which in turn will have impact on understanding how brain circuits direct behavior^20^. We posit that it would be advantageous to quantify precisely the differences in optical signals acquired with genetically-encoded calcium indicators (GECIs) versus genetically-encoded voltage indicators (GEVIs). Defined and quantified (relative amplitudes, kinetics and waveform) differences between GECI and GEVI population optical signals (if any) would strengthen the interpretation of the GCaMP-based population imaging studies^21–23^. Knowing the temporal and amplitudinal relations between the GECI signals and compound voltage transients that actually underlie these GECI signals would bring the mesoscopic GCaMP data closer to the context of neural ensembles operating through electrical (not calcium) signaling^1,24,25^.

Some may argue that the slowness of the GCaMP signal is a well-established fact and as long as this disclaimer is included in publication, we should let it pass, and preserve the equals sign between GCaMP optical signals on one side and neuronal activity on the other side of the equations. Perhaps a better approach would be to know precisely the discrepancy between the waveform of the GCaMP optical signal and the waveform of the voltage event occurring in the network. What errors do GCaMP experiments systematically introduce into our understanding of population electrical signaling in the brain^21,26^?

Here we use two transgenic animals, expressing genetically-encoded indicators in cortical pyramidal neurons. Brain slices harvested from these two animal strains were subjected to an identical experimental paradigm (patterned synaptic stimulation), which allowed us to characterize the differences in optical population signals, GECI versus GEVI. Our quantifications will be useful for interpreting calcium imaging data void of cellular resolution—GCaMP population signals. GCaMP population imaging, also known as mesoscopic imaging, is becoming increasingly popular, due to affordable equipment, excellent signal to noise ratio (SNR), and commercially-available GCaMP genetic encoding kits, which provide means of measuring activity codes in defined cell types across the entire brain. GCaMP macroscopic imaging techniques (mesoscopic, volume, population, fiber photometry) emerge as essential tools for testing how intact brain dynamics vary across diverse behaviors. In the current study we evaluate the ability and veracity of GECIs in reporting the occurrence of compound depolarization signals in neocortical layer 2/3 neuropil.

## Results

### GEVI and GECI transgenic animals

**Figure 1:**
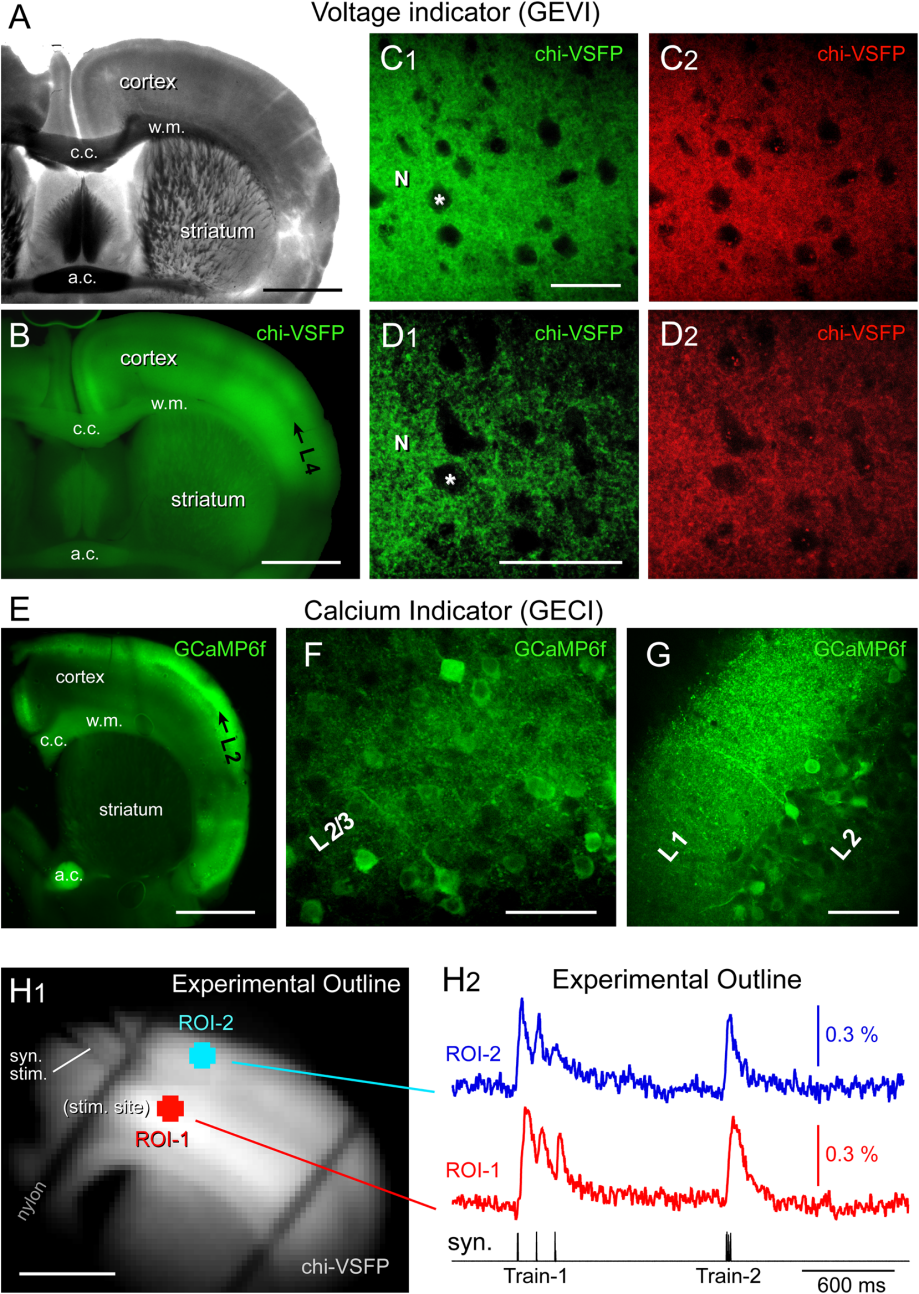
Transgenic animals. (**A**) Transgenic animal expressing a GEVI variant called “chi-VSFP”. P151 days, Male. A coronal section captured in transmitted light. Scale, 1 mm. (**B**) Same brain section as in (**A**), wide-field fluorescence (GFP filters). Black arrow marks cortical layer 4 (L4). “c.c.”—corpus callosum; “w.m.”—white matter; “a.c.”—anterior commissure. (**C1**) Confocal image of the somatosensory cortex (layer 2/3) in green channel (excitation 488 nm, emission 510–545 nm). “N”—marks neuropil. “Asterisk”—marks neuronal cell body. Scale, 50 µm. (**C2**) Same as in (**C1**) except the emission window is now 578–625 nm. (**D1**,**D2**) Same as in (**C1**,**C2**) except the location is cortical layer 5. Scale, 50 µm. (**E**) Transgenic animal expressing a GECI variant called “GCaMP6f”. P254 days, Male. (**F**) Confocal image of the somatosensory cortex (layer 2/3) in green channel (excitation 488 nm, emission 510–545 nm). Scale, 50 µm. (**G**) GCaMP-positive neuropil in cortical layer 1. (**H1**) One video frame obtained with data acquisition camera (80 × 80 pixel) during a voltage imaging trial (cropped). Transgenic animal expressing chi-VSFP, age P210 days, Female. Scale, 1 mm. Syn. Stim—marks a glass stimulation electrode entering the brain slice. (**H2**) Synaptically-evoked optical signals from two regions of interest (ROIs). ROI-1 is at the stimulation site, in cortical layer 5. ROI-2 is selected form L2/3. Each trace is product of temporal averaging (4 sweeps), low-pass filtering (40 Hz cutoff, Gaussian), bleach subtraction (exponential fit) and high-pass filtering (Tau Filter = 10). Synaptic stimulation *Train-1* consists of three electrical pulses at 120 ms ISI, while *Train-2* employs a 12 ms ISI.

In brain slices harvested from GEVI transgenic mice (Fig. 1A), cerebral cortex was examined for expression of fluorescent indicator chi-VSFP (Fig. 1B). The voltage indicator was expressed in all pyramidal neurons, consistent with CaMK2A-tTA;tetO-chiVSFP genotype, and consistent with the findings of the T. Knopfel group who initially developed this animal line^27^. A dense neuropil composed of fluorescent dendrites and axons was found in layer 2/3 (Fig. 1C1) and layer 5 (Fig. 1D1). When excited by 488 nm light, the voltage indicator chi-VSFP glowed in green channel, emission 510–545 nm (Fig. 1C1,D1), but also in the red spectra, emission 578–625 nm (Fig. 1C2,D2). This dual emission of chi-VSFP is due to the presence of two fluorophores in each indicator molecule^28^.

In brain slices prepared from the GECI transgenic mice (Rasgrf2-dCre;CaMK2A-tTA; TITL-GCaMP6f), low magnification images documented the expression of fluorescent calcium indicator GCaMP6f (Fig. 1E). In this animal line, calcium indicator GCaMP6f was densely expressed in all pyramidal neurons of layer 2/3, hence a very dense fluorescently-labeled neuropil (Fig. 1F). When excited by 488 nm light, GCaMP6f glowed in green channel (Fig. 1F), but not in red (not shown). In the GEVI mice, the cell bodies were represented as dark holes in a “wall of fluorescence” (Fig. 1C1,D1, asterisk). In the GECI mice, on the other hand, the cell bodies of cortical pyramidal neurons were readily distinguishable in images, popping out from the background fluorescence, together with proximal apical dendrites (Fig. 1F). Difference in the confocal images from a GEVI mouse versus a GECI mouse is due to the GEVI being a plasma membrane sensor while the GECI is a cytosolic sensor. Concentration of the GECI fluorescence to the cell body is a clear advantage in spatially-resolved imaging applications^29^, because it is far easier to see which cell bodies are responding to a calcium signal (Fig. 1F) than to determine the voltage signal source from dense fluorescent neuropil (Fig. 1D)^30^.

The extent of the GCaMP6f expression in dendrites^31^ and axons is best appreciated in cortical layer 1. Neocortical layer 1 is largely void of neuronal cell bodies. A few scattered GABAergic neurons inside L1 were not labeled by indicator, hence the fluorescence yield predominantly emanates from dendrites and axons of excitatory pyramidal neurons (Fig. 1G). Henceforth, all quantitative statements such as “GECI’s have x-time better signals than GEVI’s” should apply to the specific GEVI (chi-VSFP) and specific GECI (GCaMP6f) studied in this report.

### Experimental outline

Experiments were performed in acute brain slices using standard electrophysiological procedures^32^. The experimental paradigm was based on two trains of synaptic stimulation, delivered via a monopolar glass electrode (2 MΩ) positioned in one cortical layer (Fig. 1H1). Synaptic stimulation consisted of two triplets of current pulses with 1 s window between the two trains (Fig. 1H2, black trace, “syn.”). “*Train-1*” employed 3 pulses at 120 ms inter-stimulus interval (ISI) (8.3 Hz), and “*Train-2*” employed 3 pulses at 12 ms ISI (83 Hz). This synaptic stimulation paradigm, with fixed stimulus current intensity (135 nA) and fixed individual pulse duration (1 ms), was used in all experimental measurements in both GEVI and GECI transgenic animals. All displayed traces are products of spatial averaging from multiple pixels (5–21) inside a given ROI (Fig. 1H). The GEVI (chi-VSFP) signals recorded at a green emission filter (535/50 nm) have negative polarity in our raw data records. However, all optical signals shown in this and the following figures have been inverted in display. We feel that inverted GEVI optical signals (positive with depolarization) are more appropriate for presentations especially if comparisons are made against the GECI optical signals, which also are positive with depolarization.

### Temporal dynamics of optical signals in GECI and GEVI measurements

Over experiments performed in individual neurons co-labeled with GECI and GEVI, it is well known that GEVI signals are faster than the GECI optical signals^9,33,34^. How do these differences (in individual neuron optical signaling) transfer to the population imaging method, lacking cellular resolution? In the current study, mixed synaptic and action potentials were evoked by standard synaptic stimulation routine, and optically recorded from hundreds of dendrites and axons encompassed within the same ROI. In response to extracellular electrical stimulation, neurons respond with a range of the onset-timings and depolarization amplitudes and waveforms. Neurons closer to the stimulation electrode experience higher electrical field from the stimulation pulse. Also, large diameter axon fibers are more easily activated by extracellular electrical stimulations than smaller fibers^35^. Such desynchronized activations of hundreds of neurons, and ensuing desynchronized slow membrane depolarizations (EPSPs), are expected to blur the differences between GECI and GEVI. Compound signal emerging from multiple dendritic and axonal branches, belonging to hundreds of different neurons, distributed at several focal planes inside brain slice (all these structures projecting their light onto the same optical detector) would further blur the difference between GECI and GEVI population signals. Is it possible that brain population signals are equally well represented in population GECI and population GEVI measurements?

**Figure 2:**
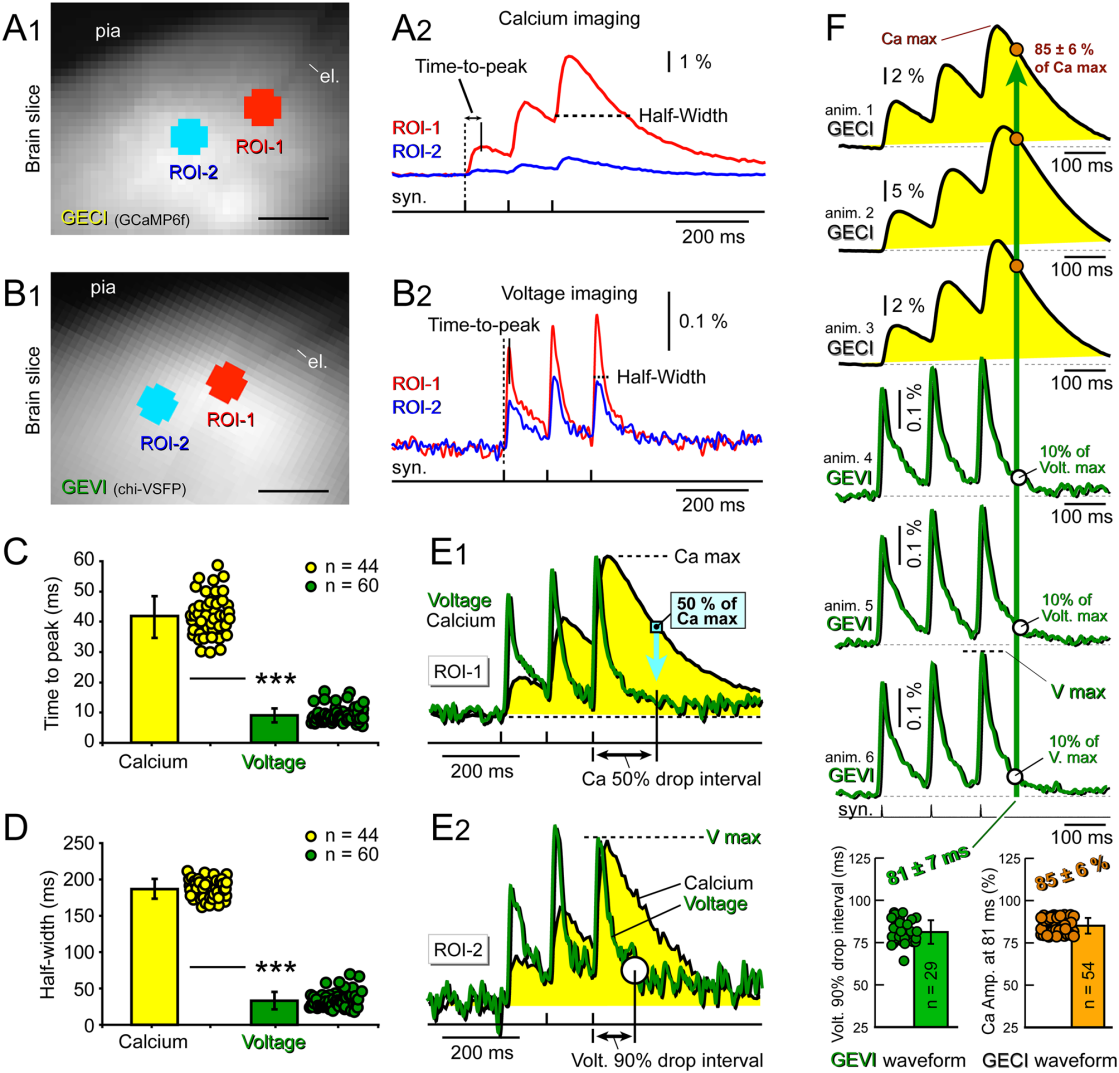
Onset, duration and decay—calcium (GECI) versus voltage (GEVI) optical signals. (**A1**) Transgenic animal expressing GECI (GCaMP6f) in cortical pyramidal neurons. Coronal brain slice imaged by NeuroCCD 80 × 80 pixel. Scale, 200 µm. Two ROIs are selected, at the stimulation site (ROI-1), and 200 µm away from the stimulation site (ROI-2). (**A2**) Synaptically-evoked optical signals in GECI mouse. Time-to-peak parameter is measured between stimulus pulse and calcium signal peak. Signal duration is measured in the 3rd peak at half amplitude (*Half-Width*). (**B1**) Same as (**A1**), except the transgenic animal is expressing GEVI (chi-VSFP). (**B2**) Same as in (**A2**), except GEVI mouse. (**C**) Each dot represents one measurement (one experimental trial) of the time-to-peak parameter quantified at the stimulation site (ROI-1). Calcium: 44 trials, in 6 brain slices from 3 animals. Voltage: 60 trials, in 13 slices, of 7 animals. ***, p < 0.0001. (**D**) Same as in (**C**), except different parameter, half-width. (**E1**) Calcium (black) and voltage (green) optical signals are superimposed on the same time scale. Ca signal decreases 50% in time interval marked by double arrow. Vertical turquois line marks the time point “144.5 ± 10.5 ms”, when, on average, Ca signal amplitudes dropped down to 50% of their maxima. At that moment of time, “144.5 ± 10.5 ms”, Ca signal is at 50%, while the voltage trace is near the baseline (0–5%). (**E2**) Same as in (**E1**), except different ROI (away from the stimulation site, ROI-2). Voltage signal decreases by 90% in time interval marked by double arrow. (**F**) Three optical signals from 3 GECI animals, followed by 3 optical signals from 3 GEVI animals. Optical signals are aligned by synaptic stimulation pulses (syn.). Bottom: Green data points: time intervals (from the synaptic stimulus) at which voltage signals dropped down to 10% of their maxima (*V max*). The average time delay for this 90% descent was 81 ± 7 ms (n = 29). Vertical green arrow marks the time point “81 ± 7 ms” transecting voltage and calcium waveforms. Bottom, orange points: Ca signal amplitudes measured 81 ms after the syn. pulse; normalized to the “*Ca max*” of the same trace; 85 ± 6% (n = 54). The voltage had dropped down to 10%, while the calcium is still lingering at ~ 85%.

To answer this question we quantified the optical signal rise time and optical signal duration at half amplitude (half-width) in GECI vs GEVI brain slices. These measurements were performed in brain slices bathed with standard saline (no drugs). Briefly, brain slices were imaged with a 10 × water immersion lens (NA = 0.30) positioned on the cortical layer 2/3, where we documented high density of indicator-positive pyramidal neurons in both GECI and GEVI animal strains (Fig. 1), resulting in a complete loss of neuronal individuality. Optical signals were measured simultaneously at the stimulation site (Fig. 2A1,B1, ROI-1) and 200 µm away from the stimulation site, in the same cortical lamina L2/3 (ROI-2). Each ROI contained 21 pixels, collectively encompassing a surface area of 0.0084 mm^2^. Each optical trace displayed in Fig. 2A2,B2 is a spatial average of 21 individual pixels and temporal average of 4 sweeps, with more than 10 s of wait time between these sweeps. Spatial averaging (21 pixels) and temporal averaging (4 sweeps) were performed to improve the SNR in optical measurements.

We used the same stimulation pulse duration (1 ms) and current pulse intensity (135 nA) in all GECI and GEVI measurements for testing optical signal rise time and optical signal duration (n = 104). Both GECI and GEVI signals showed notable amplitude decline away from the stimulation site (Fig. 2A2,B2, compare ROI-2 to ROI-1). At the stimulation site (ROI-1), the optical signal rise (time-to-peak) was quantified for the first synaptic event in *Train-1*, because this event rises from a stable baseline, unlike the later events (2nd and 3rd). The parameter “time-to-peak” was defined as time interval from the onset of the stimulus pulse to the peak of the optical signal (Fig. 2A2,B2, *Time-to-peak*). In GECI animals (3 animals, 6 brain slices) we acquired 44 experimental traces using the *Train-1* synaptic stimulation paradigm (3 pulses at 120 ms ISI). The average time-to-peak value in calcium population imaging of synaptically-evoked signals (n = 44) was 41.9 ± 6.8 ms (mean ± stdev, Fig. 2C). The average time-to-peak value in voltage population imaging was only 9.05 ± 3.56 ms (60 recordings in 13 slices from 7 animals, Fig. 2C). The GECI signal’s rise dynamics (time-to-peak) was statistically significantly slower than that of the GEVI signals (Fig. 2C, ***). On average, voltage peaks occurred ~ 30 ms sooner than the calcium peaks in synaptic stimulation trials.

Quantifications of signal duration were performed at the half amplitude of the 3^rd^ event (Fig. 2A2,B2, half-width), because the decay phase of the 3rd event was undisturbed by subsequent overlapping events (the 3rd event was the last event in train). Superposition of time-aligned GECI and GEVI traces revealed large differences in signal duration (half-width), caused by slower decay times of the GECI signals, and these differences persisted at the synaptic stimulation site (Fig. 2E1), as well as 200 µm away from the stimulation site (Fig. 2E2). The average half-widths of the GECI and GEVI optical signals at the stimulation site (ROI-1) were 187.1 ± 13.3 ms and 33.4 ± 10.9 ms, respectively, Fig. 2D, statistically significant difference, p < 0.0001. On average, the rise time of the GECI signal was ~ 4 times slower (Fig. 2C) and the duration of the GECI signal was ~ 6 times longer (Fig. 2D) than the duration of the corresponding GEVI signal.

Synaptically-evoked optical signals from the GECI and GEVI animals, aligned in time (Fig. 2F) showed differences in temporal dynamics. That is, at the same point of the trial’s time course, the population voltage signal is largely gone (almost returned to the baseline), while the population calcium signal is still on the upsurge. Using synaptic stimulus as a reference time point (0 ms), we measured the amount of time the GEVI optical signals required to drop by 90% from their peak values, *V max* (Fig. 2F, bottom graph). On average, 81 ms after the onset of the 3rd synaptic stimulus, the GEVI optical signals repolarized by 90% (81 ± 7 ms, mean ± stdev, n = 29, Fig. 2F, green bar). We then measured the relative amplitude of the GECI signals at the equivalent experimental point, 81 ms after the onset of the 3rd synaptic stimulus (Fig. 2F, green vertical arrow). In the 81st millisecond, the GECI optical signal was on average 85% of its maximal value (Fig. 2F, *Ca max*). In the time point in which voltage dropped down to 10% of its maximum (*V max*), the calcium signals were still near their peaks, 85 ± 6% of the *Ca max* (mean ± stdev, n = 29, Fig. 2F, orange bar).

Finally we measured the amount of time for Ca signal to lose 50% of its maxima (Fig. 2E1, “*Ca 50% drop interval*”). On average, it takes 144.5 ± 10.5 ms (mean ± stdev, n = 54) for GCaMP6f population signals to decay by 50%. In Fig. 2E1, the timing of the turquois downward arrow is 144 ms after the onset of the synaptic stimulus, and this arrow points to very low relative amplitudes of voltage signals (voltage is near the baseline). In summary, measurements performed with GECIs and GEVIs, indicate that when neuronal population is near-completely repolarized (down to only 0–5% of the *V max*), the corresponding GCaMP6f signal is still relatively strong, at half amplitude (50% of the Ca max, Fig. 2E1).

### Traveling signals in GECI and GEVI measurements

One of the most exciting features of the multi-site voltage imaging technique is its capacity for monitoring the propagation of depolarization waves. As a depolarization signal (e.g. action potential) propagates from one cellular compartment to another, voltage imaging optical signals report the speed of the travel, and minute changes in voltage waveforms, as the propagating signal encounters compartments with various passive and active properties. The compartment to compartment travel time (latency), and compartment-to-compartment changes in voltage waveform, are regularly accomplished in voltage imaging experiments^36,37^, but mostly evaded researchers in similar calcium imaging experiments^37,38^.

**Figure 3:**
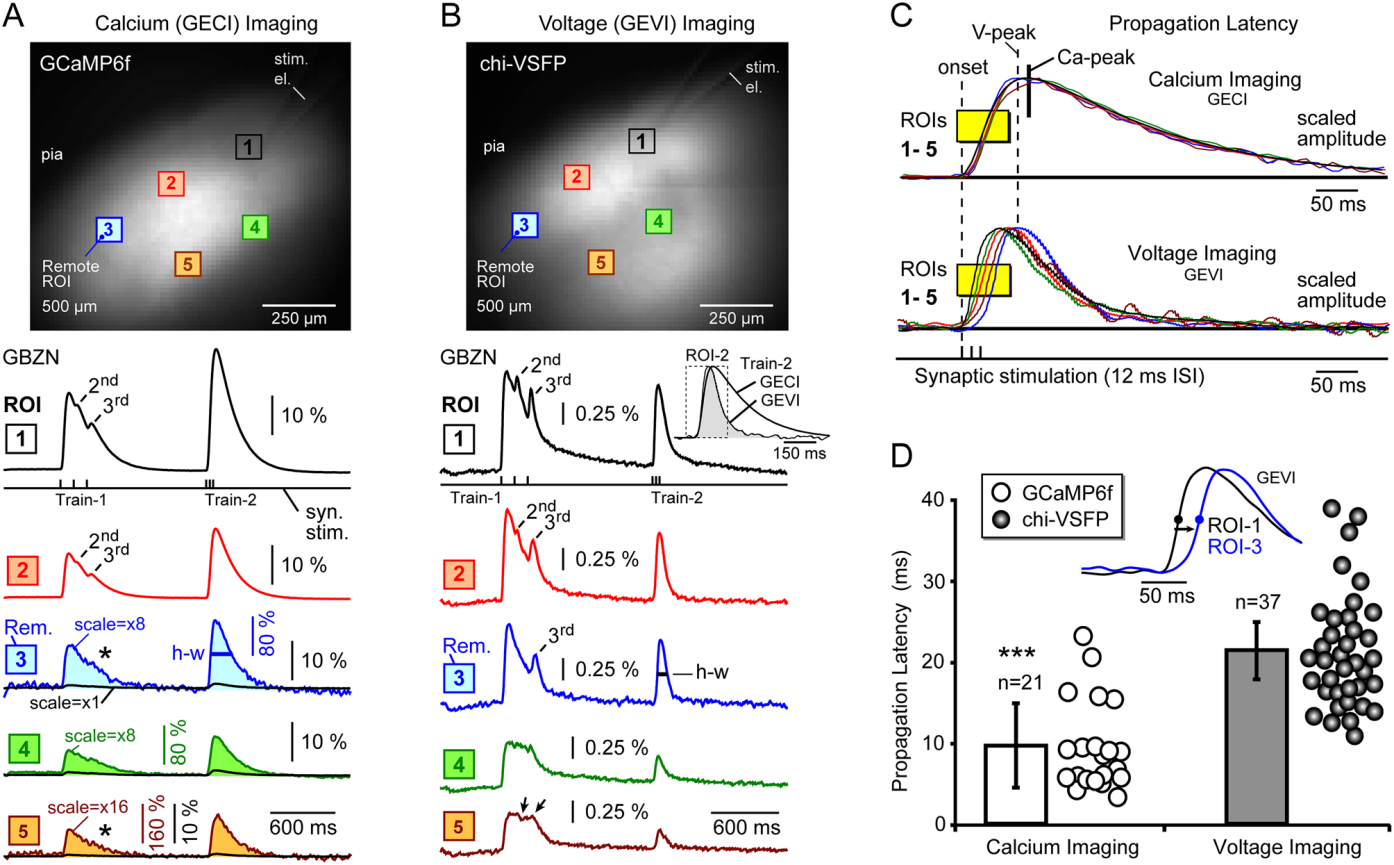
Optical signal propagation—calcium vs. voltage imaging. (**A**) Top, image: Surface of a cortical brain slice prepared from GCaMP6f animal, with ROIs and glass electrode for synaptic stimulation. Bottom, traces: Synaptically-evoked calcium (GECI) transients in the presence of GABA-A receptor antagonist, gabazine, GBZN [10 µM]. Synaptic stimulation comprised two triplets, 8.3 Hz (*Train-1*) and 83 Hz (*Train-2*), respectively. Optical traces were recorded simultaneously from 5 ROIs. The remote traces (ROI-3-4-5) are shown at two amplifications: × 1 (black) and × 8, × 16 (color). Asterisk marks uneventful waveform. (**B**) Same as in (**A**), except the brain slice was prepared from a chi-VSFP animal. Black arrows mark fast changes in GEVI waveforms, which are unmatched in the GECI records (asterisk). Inset: Differences in the peak timing and signal duration between a GECI and GEVI trace at ROI-2. (**C**) Traces from (**A**) and (**B**) (*Train-2*) are amplitude-scaled, time-aligned, and superimposed on a faster time scale. Yellow box on the rising slopes of traces indicates the amplitude level (half-amplitude) at which latencies were quantified. Note that voltage transients (bottom) show a greater variety of signal latencies compared to the calcium transients (top). Vertical dashed line “onset” marks the onset of synaptic stimulation. Vertical dashed line “*V-peak*” marks the peak of the most delayed voltage transient (ROI-3). Thick vertical line “*Ca-peak*” marks the peak of the GCaMP6f transients. (**D**) Inset: Propagation latency between two ROIs was measured at half-amplitude. White bar is an average latency measured at 500 µm away from the stimulation site, obtained in 21 recordings from 6 brain slices treated with gabazine, in 3 GCaMP animals (mean ± stdev). Gray bar is an average 500 µm latency obtained in 37 recordings from 15 GBZN-treated brain slices, in 6 chi-VSFP animals, p < 0.00001.

We hypothesized that synaptically-evoked population signals propagate across the brain slice parenchyma, and on their way, these signals encounter various cells, synapses, dendrites and axons, which may generate small propagation latencies and small changes in signal waveform. Is it possible that mixing of signals from hundreds of neurons projecting to the same optical detector (e.g. ROI) would render GECI and GEVI traces very similar, in many aspects? To answer this question, we characterized the propagation of synaptically-evoked population signals in brain slices, using both GECI and GEVI imaging modalities. All experiments in this experimental series were performed with synaptic stimulation electrode positioned in cortical layer 2/3, and slices were bathed in GABA-A receptor antagonist, gabazine [10 µM], to allow for stronger propagation of depolarizing signals. We used the same stimulation pulse duration, current intensity, pulse frequency and stimulation electrode resistance (2 MΩ) in GECI and GEVI measurements (n = 58 optical trials). Figure 3A,B show an identical experimental trial performed in GECI and GEVI animal lines, respectively, with 5 regions of interest (ROI) selected across layers 2/3 and 5. Optical signals belonging to the same experimental trial are displayed on the same amplitude scale, 10% ΔF/F scale-bar for GECI (3A), and 0.25% ΔF/F scale-bar for GEVI (3B). On the first glance, GECI signals exhibit a severe distance-dependent amplitude decline, significantly more pronounced than the GEVI signals (Fig. 3, compare the black (scale =  ×1) traces of A versus the colored traces of B). In the GECI trial, the ROIs at the stimulation site (ROI-1) showed ~ 20-fold greater amplitude than the GECI trace at remote ROIs (Fig. 3A, ROI-3, black trace). Amplitude blow-up of the black GECI traces by ×8 or ×16 times (Fig. 3A, ROI-3, ROI-4, and ROI-5, colored traces) showed that attenuated calcium traces still contain legible waveforms with plenty temporal clues including: the slope of rise, timing of the signal peak, signal half-width, and the rate of decay. In GEVI experiments (Fig. 3B), the relative amplitude decline with distance from the stimulation was less pronounced, with the ROIs at the stimulation site (ROI-1) showing only a twofold greater amplitude than the remote ROIs (ROI-3, 4 and 5). At the stimulation sites, the synaptic *Train-1* is represented by 3 peaks in the optical signal for both GECI (Fig. 3A, ROI-1) and GEVI measurements (Fig. 3B, ROI-1) (n = 58). At remote sites (ROI-3), however, these late peaks (e.g. 2nd or 3rd) were depleted in GECI (asterisk), but still persisted in the GEVI recordings (Fig. 3A,B). The synaptic *Train-2* was represented by a single peak (optical signal) for both GECI (Fig. 3A) and GEVI measurements (Fig. 3B), regardless of the distance from the stimulation site (ROIs 1–5). These data (n = 58) indicate that at certain frequency of synaptic events (e.g. 83 Hz (12 ms ISI)) the current population optical signals cannot resolve individual synaptic events (3 events) in neither GECI nor GEVI modality. Nevertheless, owing to a much faster decay of the GEVI optical signal, the half-width of the optical response to synaptic *Train-2* was markedly shorter in GEVI compared to GECI experiments (Fig. 3B, inset). The GECI optical signal was wider than the corresponding, distance-matched GEVI optical signal, regardless of the ROI’s distance from the stimulation site (Fig. 3A,B, h-w). At any given distance from the synaptic stimulation site, the GECI transient was ~ 2.3 times wider than the counterpart GEVI transient (n = 12 GECI and 12 GEVI measurements). Both GECI and GEVI waveforms experienced changes as depolarizing signal propagated through slice, but GEVI waveforms regularly contained greater number of interesting features (peaks and troughs) than the GECI waveforms (n = 58).

Next we focused on the signal propagation latency. To illustrate propagation latency, the traces from 3A and 3B were scaled to the same amplitude in Fig. 3C. These recordings were time-aligned based on the onset of the first synaptic pulse marked by the vertical dashed line “onset” (Fig. 3C). While all calcium signals reached their peak approximately at the same moment of time (“Ca-peak”), and their rise phases more-or-less aligned on top of each other, inside a short window of time, smaller than 10 ms (Fig. 3C, Calcium Imaging, yellow box), the voltage traces on the other hand, showed peaks distributed in time, and their rising phases were also spread over a window of time greater than 30 ms (Fig. 3C, Voltage Imaging, yellow box). Interestingly, the peak of the most delayed voltage transient (vertical dashed line “*V-peak*”) often preceded the peak of the fastest calcium transients (Fig. 3C, “*Ca-peak*”). We quantified propagation latency between optical signals in brain slices stimulated using *Train-2* synaptic stimulation paradigm (3 pulses at 12 ms ISI) and measured 500 µm apart (e.g. ROI-1 and ROI-3). Propagation latency was measured at signal half-amplitude, as depicted in Fig. 3D, inset. Paradoxically, the slower imaging modality (GECI) showed much smaller propagation latency than the faster imaging modality (GEVI). More specifically, the average propagation latency, 500 µm away from the stimulation site, for GECI trials was 9.6 ± 5.5 ms (n = 21 recordings, 6 brain slices from 3 animals, Fig. 3D, white points). The average propagation latency for GEVI trials was 21.3 ± 7.0 ms (n = 37 recordings, 15 brain slices from 6 animals, Fig. 3D, gray points). Unpaired t-test detected significant differences between GECI and GEVI propagation latency data, p < 0.00001.

### Temporal summation

**Figure 4:**
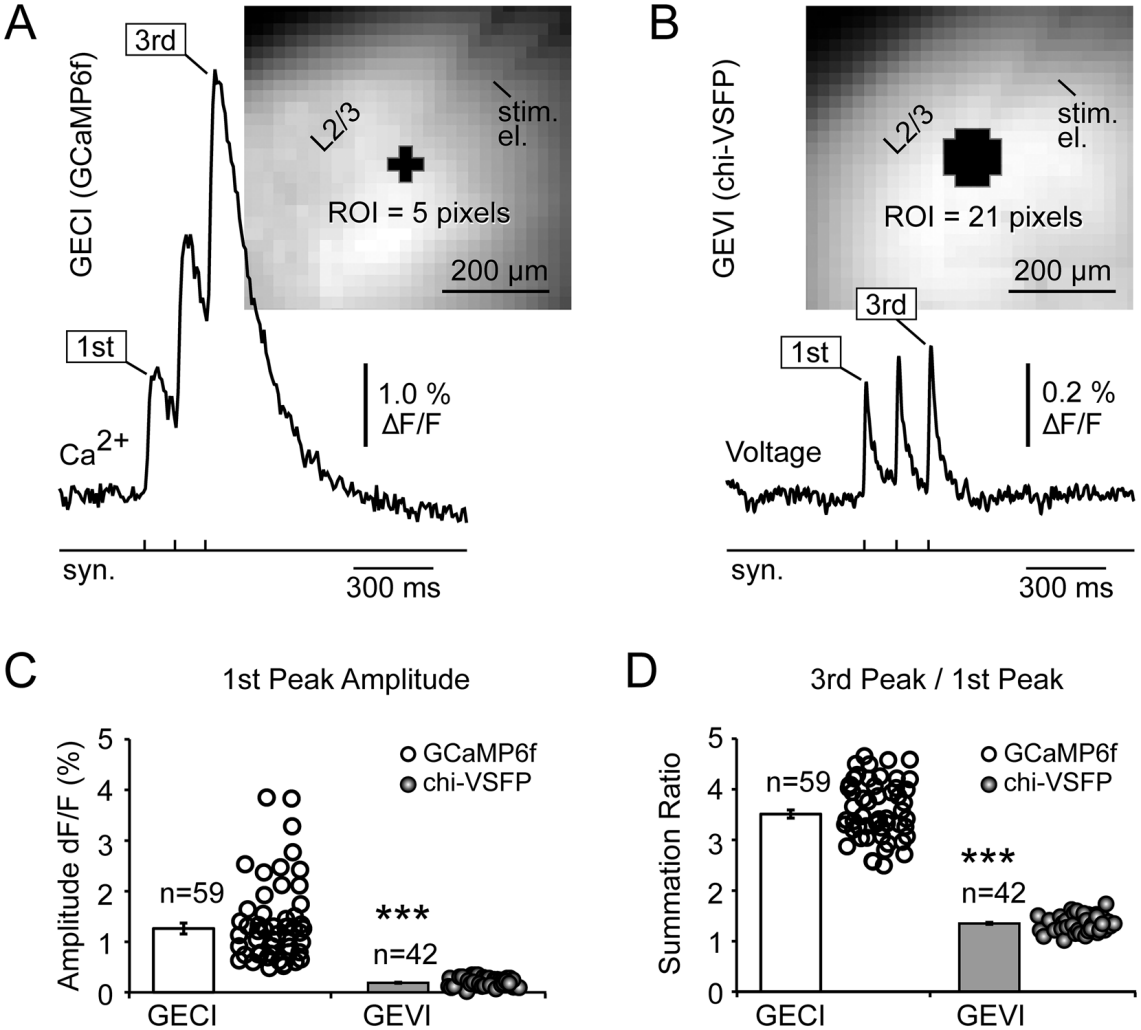
Optical SNR, amplitude (ΔF/F), and temporal summation—calcium vs. voltage imaging. (**A**) Image: Cortical brain slice harvested from GCaMP6f animal, with ROI and glass electrode (el.) for synaptic stimulation. Trace: Synaptically-evoked calcium transients obtained from the ROI (5 pixel spatial averaging). Synaptic stimulation comprised three pulses at 120 ms interval (8.3 Hz). Temporal average of 4 trials, bleach correction, 40 Hz low-pass. (**B**) Same as in (**A**), except brain slice harvested from a chi-VSFP animal, and much greater size of the ROI (21 pixels). (**C**) Amplitude (ΔF/F) of the 1st transient is represented by a raster dot plot and the color-matched bar (mean ± sem). *** indicate p < 0.00001. White (GCaMP6f): 27 recordings from 6 brain slices, in 3 GCaMP6f animals. Gray (chi-VSFP): 42 recordings from 19 brain slices, in 8 chi-VSFP animals, p < 0.00001. (**D**) Same as in ***C*** except, “summation ratio” was measured as an amplitude ratio between the 3rd and the 1st peak in the same optical trace.

In the next series of experiments we compared GECI and GEVI optical signals based on the: SNR, signal amplitude (ΔF/F), and temporal summation. All measurements were performed in brain slices bathed with standard saline (no drugs). The intensity of illumination was adjusted to produce similar levels of resting fluorescence in both GECI and GEVI slices at 1 kHz sampling rate. Similar SNR was achieved when 5 pixels were spatially averaged in GECI measurements compared to 21 pixels in GEVI measurements (Fig. 4A,B). Note that the GEVI signal shown in Fig. 4B is a spatial average of 21 pixels. We quantified SNR using 5-pixel spatial averaging, 4-sweep temporal averaging, and fully open frequency band (no filtering) for both GECI and GEVI signals. This was the minimal averaging that would allow us to resolve GEVI signals in traces with full-size high frequency noise (unfiltered). In other words, GEVI signals are so small that we cannot resolve synaptically-evoked population responses from the background noise, unless we use temporal averaging, spatial averaging, and low-pass filtering.

The 1st peak in *Train-1* was measured in both GECI and GEVI experiments. In GECI experiments, the average SNR measured at stimulation site (ROI-1) was 17.4 ± 7.49 (mean ± stdev, n = 59), compared to only 2.1 ± 0.4 (n = 42) obtained in the GEVI experiments. In summary, the SNR was on average 8 times better in GECI compared to the GEVI measurement of synaptically-evoked population signals (p < 0.00001).

For comparisons of the signal size (expressed as ΔF/F, in %) in GECI versus GEVI experiments, again we used only the first event in *Train-1* (Fig. 4A,B, 1st), temporal averaging (4 sweeps), spatial averaging (21 pixels) and low-pass filter (40 Hz cutoff). In GECI experiments (Ca Imaging), the first peak amplitude was on average 1.26 ± 0.11% ΔF/F (mean ± sem, n = 59 measurements in 6 slices of 3 animals expressing GCaMP6f). In GEVI experiments (Voltage Imaging), the first peak amplitude was on average only 0.19 ± 0.01% ΔF/F (mean ± sem, n = 42 measurements in 19 slices of 8 animals expressing voltage indicator chi-VSFP, p < 0.00001). Therefore, in population imaging with no cellular resolution, when neuronal responses are evoked by synaptic stimulation delivered in layer 2/3 (Fig. 4A,B), the GECI signal amplitudes were on average 6.6 times larger than the GEVI amplitudes (Fig. 4C).

Temporal summation in optical signals was evaluated by measuring the 3rd and 1st peak (Fig. 4A,B, 3rd and 1st peak), and then calculating 3rd/1st amplitude ratio for each experimental trial. In GECI experiments (Ca Imaging), the 3rd to the 1st peak amplitude ratio was on average 3.51 ± 0.08 (mean ± sem, n = 59 measurements in 6 slices of 3 GCaMP6f animals, Fig. 4D). In GEVI experiments (Voltage Imaging), the 3rd to the 1st peak ratio was on average 1.35 ± 0.02 (mean ± sem, n = 42 measurements in 19 slices of 8 animals expressing voltage indicator chi-VSFP, p < 0.00001). These measurements indicate that in population imaging with no cellular resolution, when neuronal responses are evoked by synaptic stimulation delivered in layer 2/3, at 120 ms interval, 8.3 Hz (Fig. 4A,B), the GECI transients show on average 2.6 times stronger temporal summation than the GEVI transients on the same task (Fig. 4C). Since GECI transients have 6.6 times stronger signals and they summate 2.6 times greater than the corresponding GEVI signals, as a result, the ΔF/F amplitude of the 3rd peak in GECI imaging experiments was on average ~ 17 times higher than the ΔF/F amplitude of the 3rd peak in GEVI measurements (compare 4A and 4B, note different ΔF/F amplitude scales).

### Amplitude attenuation with distance

**Figure 5:**
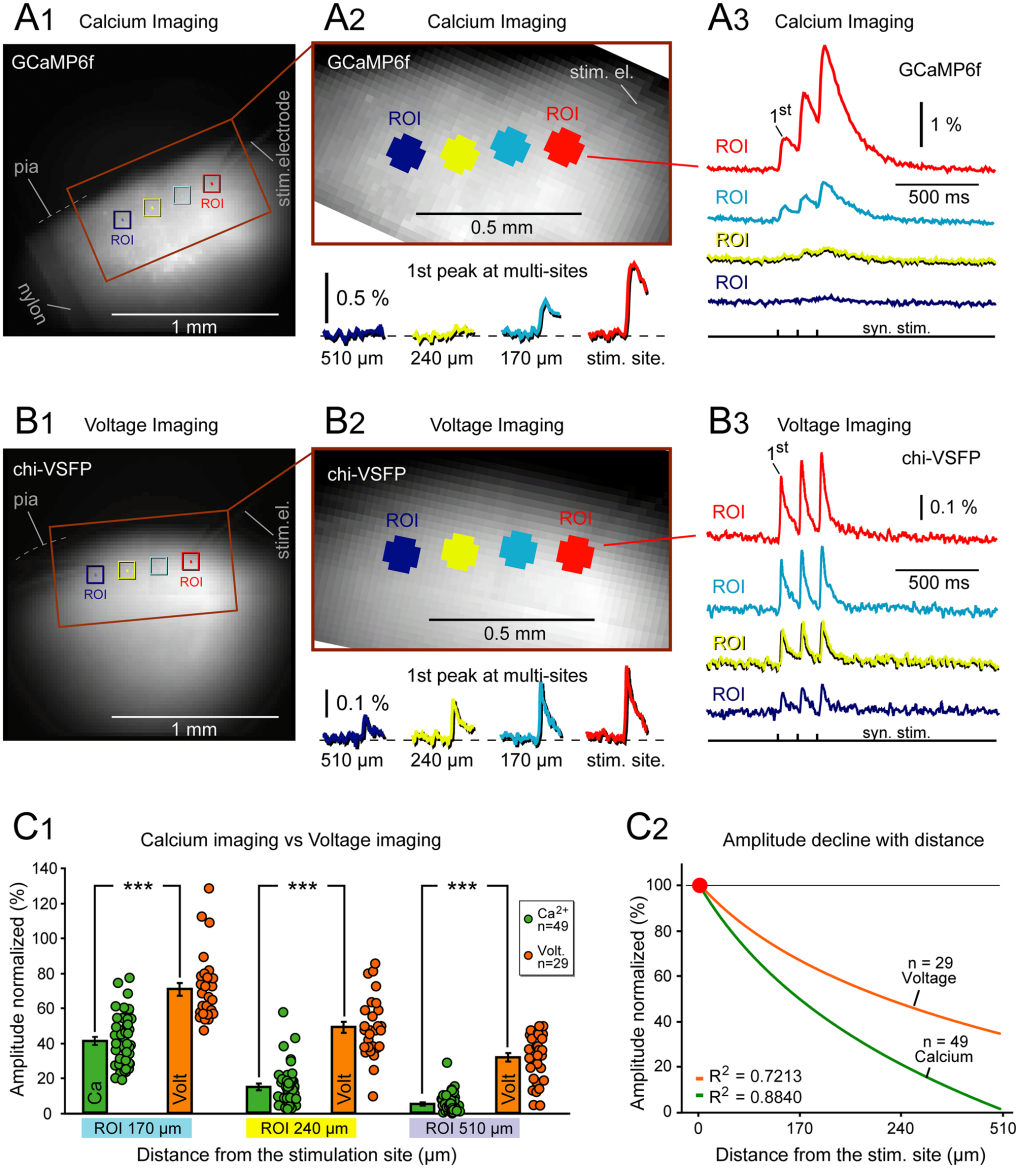
Attenuation of optical signals with distance from the stimulation site—calcium vs. voltage imaging. (**A**) A brain slice from transgenic animal expressing GCaMP6f in cortical pyramidal neurons. Synaptic stimulation electrode (el.) is positioned in cortical layer 2/3. (**B**) Blowup of the area marked by rectangle in (**A**). (**C**) Calcium signals recorded simultaneously from four ROIs marked by color in (**B**). Each trace is a spatial average of 21 pixels and temporal average of 4 trials. The first signal peak (response to the first synaptic pulse) is displayed on a greater scale below the image in (**B)**. Signals at 170, 240 and 510 µm, from the stimulation site, are shown on identical amplitude and time scales. *DEF* Same as *ABC*, except the transgenic animal expresses chi-VSFP in cortical pyramidal neurons. (**G**_**1**_) Signal amplitudes (response to first synaptic pulse) were normalized against the amplitude obtained at the stimulation site (red ROI). Each dot represents one recording. Calcium: n = 27 traces, 5 slices, 3 animals. Voltage data: n = 29 traces, 9 slices, 6 animals. Bars represent mean ± SD. ***, p < 0.0001. (**G**_**2**_) Data points shown in *G1* were fitted with logarithmic function and the trends are displayed. Distance “0 µm” is at the stimulation site (red ROI).

In both GECI and GEVI measurements of synaptically evoked signals, the signal amplitude declined with distance from the stimulation site (Figs. 2 and 3). To quantify this rate of decline we performed simultaneous measurements at 4 ROIs, located inside the cortical layer 2/3 (Fig. 5A1,B1). More specifically, the referent ROI was positioned at the stimulation site (ROI red), while the remaining ROIs were positioned along a line parallel to the pia, at 170, 240 and 510 µm away from the stimulation site (turquois, yellow and blue, respectively, Fig. 5A2-3,B2-3). All measurements were performed in brain slices bathed with standard saline (no drugs). All amplitudes were normalized against the signal amplitude at stimulation site (ROI red) of the same experimental trial. In GECI experiments (Ca Imaging), the mean (± sem) relative amplitudes at 170, 240 and 510 µm away from the stimulation site were: 41.6 ± 2.0%; 15.2 ± 1.5%; and 5.4 ± 0.74% (n = 49, Fig. 5C1, green data points and green bars). In GEVI experiments (Voltage Imaging), the mean relative amplitudes at 170, 240 and 510 µm away from the stimulation site were: 70.6 ± 3.6%; 48.9 ± 3.1%; and 31.7 ± 2.5% (n = 29, Fig. 5C1), orange data points and orange bars. The trends calculated through data points of one group (Fig. 5C2) suggested a much faster decline of the GECI signals (green line) compared to the GEVI signals (orange line). The best-fit equation for GECI signals (green trend line) was y = − 0.654 ln(x) + 0.9607, where y stands for normalized amplitude in %, and x is distance from the stimulation site in micrometers. The best-fit equation for GEVI signals (orange trend line) was y = − 0.467 ln(x) + 1.0168.

At each of the 3 distances examined in this experimental series, 170, 240 and 510 µm, the relative amplitudes of the GEVI signals were significantly (p < 0.00001) greater than those in the GECI measurements (Fig. 5C1, ***). At distance 510 µm away from the stimulation site (ROI blue), the GECI signal has lost ~ 95%, while the GEVI signal has lost ~ 60% of its initial amplitude established at the stimulation site (0 µm). This data set introduces a small paradox. Intuitively, one expects stronger signals to propagate further from the stimulation site. However, our data show that despite significantly stronger amplitudes in GECI versus GEVI measurements (Fig. 4C), and notably better SNR in GECI measurements (Fig. 4A), the GECI signals exhibit stronger distance-dependent relative amplitude decline, as they propagate through brain slice parenchyma, than the GEVI signals do (Fig. 5C1,2).

## Discussion

Using brain slices prepared from two transgenic mouse lines, and employing identical experimental paradigm (synaptic stimulation of layer 2/3) and the same equipment (same microscope, same lens, same illumination source, same optical filter set, same optical detector), we compared the performances of GECI and GEVI on the plane of synaptically-evoked cortical depolarizations in neocortical layer 2/3, as they propagate from the synaptic stimulation site through the brain parenchyma of layer 2/3. In living animals (i.e. in vivo recordings), optical signals are compromised by many contaminants, including the heart rate, breathing, non-specific mechanical vibrations (motion artifacts), sensory inputs, neuromodulatory inputs, and distinctive brain states^39,40^. None of these problems exist in vitro. Brain slice preparation provides a pointedly better setting for testing elementary optical properties of functional indicators than the in vivo brain does.

### Population imaging

The term “population imaging” is normally used for a type of an imaging study lacking single-cell resolution (e.g. wide-field imaging), where optical signal represents a “mean” response of many neurons. In population imaging experiments, activity of many elements is mixed into one representative signal; a signal that represents a given population^21,22,41–45^. The term “multi-cell imaging”, on the other hand, is a more appropriate term for imaging methods which address activity of specific individual neurons, and display one neuron per one trace^6^. In multi-cell imaging applications, each recorded trace contains only information about the cell in question^6,46^, hence the term “population imaging” is not valid.

Traditionally, researchers have used a number of terms: wide-field imaging, volumetric imaging, mesoscopic imaging, mesoscale imaging, population imaging, etc. Fiber photometry uses different kind of optic components than wide-field imaging, but often falls precisely into the category of population imaging techniques^40,47,48^. The current study is an example of a population imaging approach without single-cell information (Fig. 3A,B). In the current study, single-cell resolution was lost due to several factors including: (1) dense neuron labeling with fluorescence indicators (Fig. 1), (2) indiscriminate expression of fluorescent indicator in neuronal compartments (soma, dendrites and axons, Fig. 1), (3) absence of axial sectioning (single-photon wide-field illumination), (4) low magnification + thick focal volume (10 × objective lens), (5) light scattering through brain tissue, and (6) low-pixel camera where each pixel covers area approximately 21 × 21 µm  (Fig. 2). In the current study, all experiments employed optical signals evoked by synaptic stimulation, the activity of many cells was projected to the same detector pixel, and for that reason the reported optical signals are similar to local field potentials (LFP), which are known to be dominated by synaptic potentials^25^. If we assume a severe light scattering problem in our optical measurements, where each pixel contributes up to 50% light to its first neighbor, the spatial resolution of the reported voltage imaging results (Fig. 3) can be estimated to be 50–100 µm^41^. This conservative estimate is slightly better than the spatial resolution of the LFP signal, which is 200–400 µm^25^. Most importantly, in contrast to LFP, which senses extracellular voltages and dramatically flips signal’s polarity if a depolarization wave travels under the electrode, our GEVI method senses genuine trans-membrane voltages, in which membrane depolarization is always with positive polarity, and membrane hyperpolarization is always negative, regardless of the travel itinerary.

### Genetically encoded indicators

LFP or voltage-sensitive dyes are two experimental methods which indiscriminately collect activity from any cell in a given brain area^25,41,42^. The obvious advantage of GECI and GEVI population imaging over LFP, or voltage-sensitive dye imaging, is the cell type-specific delivery of functional indicators^29,39^. In the current study, the expression of fluorescent indicators (GECI or GEVI) was restricted to excitatory pyramidal neurons in cerebral cortex^30^. The optical signal exclusively emanated from the membranes of pyramidal neurons. Contaminating signals arriving directly from interneurons, thalamo-cortical axons, and fibers projecting into cerebral cortex from brainstem (e.g. dopaminergic, serotonergic, noradrenergic), olfactory bulb, and hippocampus, were eliminated by the genetic approach.

### Distance dependent amplitude decline

Despite having significantly greater amplitudes of synaptically-evoked GECI signals (~ 7 times greater amplitudes than the GEVI signals), and despite having significantly better SNR (~ 14 times better), the GECI signals decay very rapidly with the distance from the stimulation site, more rapidly than the GEVI signals (Fig. 5). The relative contribution of action potentials (APs) to the overall optical signal may explain this finding. GECIs mostly detect APs, while being relatively blind to AMPA-mediated synaptic depolarizations. At the synaptic stimulation site, a few cell bodies and axons generate APs. At remote recording sites, 500 µm away from the stimulation site, very few postsynaptic cells generate APs. Consequently, a GECI ROI at the stimulation site detects a very strong optical signal due to the presence of APs, while at the same time the remote GECI ROI detects a very small calcium signal due to absence of APs (Figs. 2, 3 and 5). In the voltage (GEVI) imaging modality, similar to LFP technique, the signal is primarily carried by EPSPs, while APs contribute very little^25,49^. The rate of disproportionality between the stimulation site and the remote recording site is small in the GEVI approach, because voltage signals detect predominantly the EPSP-like depolarizations^25,49^, which, in the present experimental paradigm (synaptic stimulation), occur at all recording sites (stimulation site and remote sites), unlike APs which predominantly occur at the stimulation site.

### Rise and fall kinetics

Calcium indicators are notorious for their slow kinetics. The Ca^2+^ binding kinetics and Ca^2+^ buffering nature of calcium indicators reduces peak Ca^2+^ signal, prolongs temporal decay, and enlarges diffusional spread^17^. GCaMP6f has an even slower dynamics than Ca^2+^ sensitive dyes^7,18^. The decay kinetics of GCaMP variants were studied by the GCaMP inventors^7,29^, but very little is known about the compound membrane potentials changes, which underlie the compound Ca^2+^ population signals^44^. What do we know about the voltage waveforms of the events which caused GCaMP transients reported in the cerebral cortex^21,22,26^? The current study provides specific measurements of temporal and amplitudinal discrepancies between compound voltage transients (measured by GEVI) and compound Ca^2+^ transients (measured by GECI), in cortical networks. For example, we found that the GEVI versus GECI peak discrepancies are on the order of 30 ms (Fig. 3), which is a long time in neuronal computations^50^. In 30 ms, a neuronal electrical signal can travel a round trip from one brain hemisphere to another^51^. During the 30 ms time delay between GCaMP6f signal and the actual population voltage, the superficial cortical layers complete ~ 1.5 cycles of gamma band oscillations. The information from one cortical area can be extracted very rapidly (~ 20 ms) by the next^52,53^. This means that GCaMP reports “something’s happening” in a given cortical region more than 10 ms after the cortical information processing in the voltage domain has been already completed.

On a single synaptic stimulation event, the compound (population) membrane potential rises for ~ 10 ms, while the compound (population) Ca^2+^ transient takes ~ 40 ms (Fig. 2). Besides the clear peak timing distortion, the GECI-induced distortions also come from the decay phase of the GCaMP6f signal^7,18,29^. While the voltage of the last synaptically-evoked event had almost completely repolarized, 90% returned to the baseline (Fig. 2F, white dots on voltage traces) at that same moment of time, the equivalent GECI transient was still near its maximum, dwelling at 85% of its established peak (Fig. 2F, brown dots on calcium traces). The take home message is that a popular GECI imaging method reports the peak of the “activity signal” at the moment of time when the actual depolarization is completely, or near-completely, gone from the population of cortical neurons in question.

### Signal propagation

The primary goal of multi-site neuroimaging methods is information about spatio-temporal sequences of events which constitute brain functions. The researchers seek to establish which brain region, or which part of a given neural network responded first, and which responded last, on a given behavioral task^21,54^. Researchers compared performances of GECI and GEVI (GCaMP3 and VSFP) in intact cortical tissue using visual stimuli, and found that a very similar progression of preferred azimuth with cortical distance between the two maps, although the selectivity for stimulus position was sharper in GCaMP3 responses compared to VSFP responses^55^. We found that GECI optical signals measured simultaneously at multiple sites on the surface of synaptically-stimulated brain slices show a near-simultaneous rise. The leading edges of the GECI signals were regularly slow and compressed in time (Fig. 3C), which prevented researchers from discerning the temporo-spatial sequences of faster network events. On the contrary, GEVI optical signals gave us a variety of signal propagation latencies on each recording trial (Fig. 3D) that can be used to follow the path of a depolarizing signal as it propagates from its source (synaptic stimulation site, Fig. 3A,B, ROI-1) to some arbitrary location (e.g. ROIs 2–5). The GCaMP6f signals have typically undergone minimal waveform changes as depolarization signals traveled away from the stimulation site (Fig. 3A). The GEVI waveforms, on the other hand, offered a notably richer variety of waveforms (Fig. 3B), where each peak or bend may have a physiological substrate, embedded in cell morphology, membrane excitability, connectome or basic synaptic function. For this reason, GEVI signals are better suited than GECI signals for studying the brain connectome. In summary, for the same distance traveled (~ 500 µm), GECI signals attenuated ~ 20-fold, while the GEVI signals attenuated ~ twofold, on average. Also, GEVI signals displayed variable waveforms at multiple recording sites (Fig. 3B), while the GECI waveforms were rather uniform, and less eventful (Fig. 3A, ROI-3, asterisk).

#### Temporal summation

We found that GECI signals can carry false information about the amplitudes of compound (population) membrane potentials, occurring in neuronal networks. Three network responses of nearly identical amplitudes, established by GEVI imaging (Fig. 2E, green), are coded as three events of very different relative amplitudes by the GECI imaging method (Fig. 2E, yellow). This has important implications on the interpretation of in vivo GECI data. Noisy in vivo recordings are prone to miss (fail to detect) early signals (the first event in a GECI imaging experiment, Fig. 4A, “1st”). In contrast, the GEVI experiments have an even chance of detecting each event in a series (Fig. 4B), as the first synaptically-evoked network voltage event is marginally different in amplitude compared to the subsequent events (Fig. 4D, GEVI). Our data suggest that in vivo GECI results are heavily biased towards the later network events (e.g. 3rd peak), as a result of accumulation of intracellular calcium in neurons. In in vivo recordings, late network events may emerge as powerful “units of activity” (Fig. 4A, “3rd”), while the early events (Fig. 4A, “1st”), despite having identical voltage amplitudes as the late events do (Fig. 4B), may escape detection, or be assigned a lesser significance. As a result of this technical limitation, the GECI data are essentially void of preparatory electrical signals (early events), which are probably essential for understanding the cellular basis of information processing, the buildup of network depolarizations, attractor states, and other manifestations of electrical signaling, which define brain function.

#### Limitations

The advantage of voltage optical signals to investigate fast circuit dynamics has been the primary motivation for developing GEVIs^28,39^. Compared to the GEVI variant used in the present study (chi-VSFP), several new GEVI variants had recently claimed better sensitivity and faster kinetics^5,6,46,56^. Transgenic animals expressing these most recent GEVI variants are not available to us, currently. Better sensitivity and faster kinetics is extremely important for multi-cell imaging applications, where activity of individual cells is addressed cell-by-cell^5,6,46,56^. For the population imaging applications this may be of lesser importance^39^. Performance differences between GEVI variants of variable signal strength or speed, are somewhat blurred in the population imaging mode, but not entirely eliminated^57^. The use of Archon1, ASAP3, QuasAr3 and other new generation GEVIs^5,6,46,56^, may further enhance the temporal and amplitudinal discrepancies between GECI and GEVI imaging modalities, described in Figs. 2, 3, 4, 5.

#### Raw power

Under special circumstances, wide-field GEVI signals had a SNR similar to that of signals measured with wide-field GCaMP imaging^55^. In the current study, however, the GCaMP6f amplitude and SNR were exceedingly superior to those documented in the GEVI imaging trials (Fig. 4). In the absence of temporal summation, when the 1st synaptic event was used as a standard signal, our measurements indicated that the GECI’s signal amplitude was ~ 6.6 times better than that of the GEVI’s, and the GECI’s SNR was at least eight (8) times better than the GEVI’s SNR. Though, when the 3rd event was used as a standard (high frequency stimulus causing strong temporal summation), the GECI outperformed GEVI twenty (20) fold on both, the amplitude and the SNR evaluations. This exceptional raw power (signal strength) perhaps may explain the global success of GECIs in experiments performed on the brains of living animals, asking real biological questions, and not wanting to spend the entire time fiddling with equipment and indicators. It is better to record something than record nothing.

## Methods

### Animals

Brain slices were harvested from transgenic mice (age 90–360 days, both sexes) according to the animal protocols approved by the UConn Health Institutional Animal Care and Use Committee (IACUC), in accordance with ARRIVE guidelines and institutional regulations. Transgenic animal lines were donated by Thomas Knopfel (Imperial College London, UK). Triple transgenic GECI mice (Rasgrf2-dCre;CaMK2A-tTA;TITL-GCaMP6f) expressed GCaMP6f under a Cre/TetO dependent promoter. Recombinase activity of dCre (and GCaMP6f expression) was induced with 2 or 3 trimethoprim intraperitoneal injections (20 mg/kg/injection) over 7–10 days. The GEVI mice expressed chimeric voltage sensitive fluorescent protein (chi-VSFP) in all cortical pyramidal neurons (CaMK2A-tTA;tetO-chiVSFP). All animals were housed in standard conditions with free access to food and water, in a 50% dark/light cycle.

### Microphotography

Brain slices were mounted on microscope slides and photographed on Keyence Fluorescence Microscope BZ-X800 using a 2× dry objective. Confocal images were obtained on Olympus BX51 microscope equipped with a Thorlabs Confocal Laser Scanning head, 3 laser sources, and a two-channel detection module. Both green and red images were excited at 488 nm, but collected at 510–545 nm (green) and 578–625 nm (red).

### Synaptic stimulation, calcium and voltage imaging

Some of the following methods have been used in an earlier study^57^. Following a deep anesthesia with isoflurane, mice of both sexes (ages P90–P360) were decapitated. Brains were extracted with the head immersed in ice-cold saline. The saline contained (in mM) 125 NaCl, 26 NaHCO_3_, 2.3 KCl, 1.26 KH_2_PO_4_, 2 CaCl_2_, 1 MgSO_4_ and 10 glucose. Coronal slices (300 µm) were cut from the fronto-parietal cortex, incubated at 37 °C for 30 min and then at room temperature. Acute brain slices were transferred to an Olympus BX51WI upright microscope and perfused with aerated (5% CO_2_/95% O_2_) saline. All experimental measurements were performed at 34 °C. Synaptic stimulation was achieved through a computer-generated TTL pulse and stimulus isolation unit (IsoFlex, A.M.P.I., Israel). The stimulation electrodes were pulled from 1.5 mm borosilicate glass with filament (resistance ~ 2 MΩ) and backfilled with saline. Triplets of synaptic shocks (1 ms duration, 135 nA) at 120 ms inter-stimulus interval, 8.3 Hz (*Train-1*) and 12 ms ISI, 83 Hz (*Train-2*) were delivered in the same optical recording sweep, separated by a 1 s interval. Optical trials were typically 3 s of light exposure with at least 10 s interval between two consecutive sweeps. Optical signals were sampled at 1.020 ms full-frame interval (~ 1 kHz frame rate) with NeuroCCD camera (80 × 80 pixel configuration; RedShirtImaging, Decatur, GA). Both, GCaMP6f and chi-VSFP were excited using the same 470 nm light emitting diode, LED (pE, CoolLED, Andover, UK) and imaged using the same filter set: excitation: 480/40 nm; dichroic 510 nm, and emission: 535/50 nm.

### Data analysis

Optical traces were conditioned and analyzed in Neuroplex. Bleach correction was done by subtracting an exponential fit from the optical trace. Temporal averaging (n = 4 sweeps), spatial averaging (5–21 pixels), low-pass Gaussian filter with 40 Hz cutoff, and high-pass Tau filter (10), unless stated otherwise. Optical signal amplitude was measured in Neuroplex as fractional change in light intensity (ΔF/F). The optical signal rise (time-to-peak) was quantified for the first synaptic event in *Train-1*. The parameter “time-to-peak” was defined as time interval (in ms) from the onset of the stimulus pulse (recorded inside the optical data file) to the peak of the optical signal (Fig. 2A2, Time-to-peak) in *Train-1*. Quantifications of signal duration (in ms) were performed at the half amplitude of the 3rd synaptic event in *Train-1* (Fig. 2A2, half-width). Signal propagation latency was measured using the *Train-2* stimulation paradigm, between the rising phases of optical signals in ROI-1 and ROI-3, at half amplitude, as depicted schematically in Fig. 3D, Inset. Temporal summation (“summation ratio”) was quantified as the 3rd peak amplitude divided by the 1st peak amplitude, in the same optical trace (Fig. 4). Data organization, plotting and statistical testing (unpaired Student’s t-test) were done in Excel.

## Data Availability

The datasets used and/or analyzed during the current study are available from the corresponding author upon request.

## Abbreviations

Ca: Calcium
*GECI*: Genetically-encoded calcium indicator
*ROI*: Region of interest
*ISI*: Inter-stimulus interval
*GCaMP6f*: A type of a calcium indicator
*chi-VSFP*: A type of a voltage indicator
SNR: Signal-to-noise ratio
EPSP: Excitatory postsynaptic potential
sem: Standard error of the mean
stdev: Standard deviation
LFP: Local field potential
APs: Action potentials
